# Supplementary material for: Comparative multiomics analysis of cell physiological state after culture in a basket bioreactor
Source: Sci Rep. 2022 Nov 23;12:20161. doi: 10.1038/s41598-022-24687-4 (PMC9686226; doi:10.1038/s41598-022-24687-4)
Supplement: Supplementary file 1 — Supplementary Information 1. [file 41598_2022_24687_MOESM1_ESM.zip › raw data/Transcriptomic raw data/6.Enrichment/1.GO/TE1vsTC1/GSEA/TE1/TRANSLATION_FACTOR_ACTIVITY__RNA_BINDING/TRANSLATION_FACTOR_ACTIVITY__RNA_BINDING(GO_0008135).html]

Details for gene set TRANSLATION\_FACTOR\_ACTIVITY\_\_RNA\_BINDING(GO:0008135)[GSEA]


|  || Dataset | readcount.sample |
| Phenotype | sample.cls |
| Upregulated in class | TE1 |
| GeneSet | TRANSLATION\_FACTOR\_ACTIVITY\_\_RNA\_BINDING(GO:0008135) |
| Enrichment Score (ES) | 0.50720286 |
| Normalized Enrichment Score (NES) | 1.8899018 |
| Nominal p-value | 0.0 |
| FDR q-value | 0.19123095 |
| FWER p-Value | 0.494 |
Table: GSEA Results Summary

  

Fig 1: Enrichment plot: TRANSLATION\_FACTOR\_ACTIVITY\_\_RNA\_BINDING(GO:0008135)
  
Profile of the Running ES Score & Positions of GeneSet Members on the Rank Ordered List

  

| PROBE | DESCRIPTION   (from dataset) | GENE SYMBOL | GENE\_TITLE | RANK IN GENE LIST | RANK METRIC SCORE | RUNNING ES | CORE ENRICHMENT || 1 | 103241006 | LOC103241006 |  |  | 285 | Infinity | -0.0126 | Yes |
| 2 | 103232953 | LOC103232953 |  |  | 394 | Infinity | -0.0173 | Yes |
| 3 | 103235785 | LOC103235785 |  |  | 640 | 9.310 | 0.1124 | Yes |
| 4 | 103241783 | LOC103241783 |  |  | 839 | 4.783 | 0.1758 | Yes |
| 5 | 103225452 | LOC103225452 |  |  | 1425 | 2.543 | 0.1881 | Yes |
| 6 | 103243447 | EIF1 |  |  | 1639 | 2.330 | 0.2138 | Yes |
| 7 | 103230832 | LOC103230832 |  |  | 1642 | 2.329 | 0.2488 | Yes |
| 8 | 103231689 | EIF1AX |  |  | 1936 | 2.121 | 0.2678 | Yes |
| 9 | 103238755 | LOC103238755 |  |  | 2002 | 2.083 | 0.2963 | Yes |
| 10 | 103248689 | EIF2S2 |  |  | 2110 | 2.015 | 0.3220 | Yes |
| 11 | 103223253 | EIF3D |  |  | 2256 | 1.957 | 0.3451 | Yes |
| 12 | 103246094 | EIF3J |  |  | 2282 | 1.941 | 0.3733 | Yes |
| 13 | 103238230 | LOC103238230 |  |  | 2541 | 1.841 | 0.3896 | Yes |
| 14 | 103239314 | DENR |  |  | 3003 | 1.705 | 0.3947 | Yes |
| 15 | 103240745 | MTRF1L |  |  | 3686 | 1.577 | 0.3880 | Yes |
| 16 | 103236007 | EIF4E |  |  | 3961 | 1.532 | 0.3989 | Yes |
| 17 | 103229205 | EIF2S1 |  |  | 3990 | 1.528 | 0.4207 | Yes |
| 18 | 103242289 | EIF5A |  |  | 4134 | 1.510 | 0.4372 | Yes |
| 19 | 119621207 | LOC119621207 |  |  | 4336 | 1.480 | 0.4505 | Yes |
| 20 | 103223630 | EIF3L |  |  | 4369 | 1.476 | 0.4714 | Yes |
| 21 | 103217701 | EEF1B2 |  |  | 4751 | 1.429 | 0.4760 | Yes |
| 22 | 103227433 | EIF1B |  |  | 5017 | 1.399 | 0.4852 | Yes |
| 23 | 103221258 | EIF5A2 |  |  | 5293 | 1.369 | 0.4936 | Yes |
| 24 | 103230378 | LOC103230378 |  |  | 5448 | 1.355 | 0.5072 | Yes |
| 25 | 119618915 | LOC119618915 |  |  | 6824 | 1.235 | 0.4644 | No |
| 26 | 103218088 | EIF4E2 |  |  | 6975 | 1.223 | 0.4761 | No |
| 27 | 103214333 | MTRF1 |  |  | 7478 | 1.183 | 0.4715 | No |
| 28 | 103243063 | MRPL58 |  |  | 8706 | 1.104 | 0.4333 | No |
| 29 | 103234055 | EEF1G |  |  | 9040 | 1.083 | 0.4348 | No |
| 30 | 103238595 | TSFM |  |  | 10023 | 1.018 | 0.4063 | No |
| 31 | 103229755 | EIF5 |  |  | 10316 | 1.003 | 0.4083 | No |
| 32 | 103214253 | LOC103214253 |  |  | 10436 | 0.994 | 0.4180 | No |
| 33 | 103237572 | EEF1D |  |  | 11180 | 0.945 | 0.3991 | No |
| 34 | 103216769 | LOC103216769 |  |  | 11592 | 0.920 | 0.3946 | No |
| 35 | 103227893 | EIF4E3 |  |  | 12146 | 0.885 | 0.3832 | No |
| 36 | 103230196 | EIF2D |  |  | 13583 | 0.796 | 0.3310 | No |
| 37 | 103224961 | EIF1AD |  |  | 13813 | 0.780 | 0.3326 | No |
| 38 | 103249057 | MTIF3 |  |  | 13890 | 0.776 | 0.3409 | No |
| 39 | 103216767 | LOC103216767 |  |  | 15301 | 0.688 | 0.2882 | No |
| 40 | 103219915 | LOC103219915 |  |  | 17684 | 0.517 | 0.1895 | No |
| 41 | 103239325 | CUNH12orf65 |  |  | 18557 | 0.434 | 0.1571 | No |
| 42 | 103227442 | LOC103227442 |  |  | 19134 | 0.378 | 0.1370 | No |
| 43 | 103246861 | LOC103246861 |  |  | 19731 | 0.311 | 0.1151 | No |
| 44 | 103214485 | LOC103214485 |  |  | 19925 | 0.283 | 0.1107 | No |
| 45 | 103234850 | LOC103234850 |  |  | 21168 | 0.000 | 0.0552 | No |
| 46 | 103225908 | LOC103225908 |  |  | 21341 | 0.000 | 0.0475 | No |
Table: GSEA details
[plain text format]

  

Fig 2: TRANSLATION\_FACTOR\_ACTIVITY\_\_RNA\_BINDING(GO:0008135)
  
Blue-Pink O' Gram in the Space of the Analyzed GeneSet

  

Fig 3: TRANSLATION\_FACTOR\_ACTIVITY\_\_RNA\_BINDING(GO:0008135): Random ES distribution
  
Gene set null distribution of ES for
 **TRANSLATION\_FACTOR\_ACTIVITY\_\_RNA\_BINDING(GO:0008135)**
